# Potential Natural Products Against Respiratory Viruses: A Perspective to Develop Anti-COVID-19 Medicines

**DOI:** 10.3389/fphar.2020.586993

**Published:** 2021-02-17

**Authors:** Marzieh Omrani, Mohsen Keshavarz, Samad Nejad Ebrahimi, Meysam Mehrabi, Lyndy J. McGaw, Muna Ali Abdalla, Parvaneh Mehrbod

**Affiliations:** ^1^ Department of Phytochemistry, Medicinal Plants and Drugs Research Institute, Shahid Beheshti University, Tehran, Iran; ^2^ Department of Medical Virology, The Persian Gulf Tropical Medicine Research Center, The Persian Gulf Biomedical Sciences Research Institute, Bushehr University of Medical Sciences, Bushehr, Iran; ^3^ Shafa Hospital, Qazvin University of Medical Sciences, Qazvin, Iran; ^4^ Phytomedicine Programme, Department of Paraclinical Sciences, Faculty of Veterinary Science, University of Pretoria, Onderstepoort, South Africa; ^5^ Department of Food Science and Technology, Faculty of Agriculture, University of Khartoum, Khartoum North, Sudan; ^6^ Influenza and Respiratory Viruses Department, Pasteur Institute of Iran, Tehran, Iran

**Keywords:** antiviral potential, coronavirus, COVID-19, epidemiology, mode of action, 2019-nCoV, phylogenetic, phytochemicals

## Abstract

The emergence of viral pneumonia caused by a novel coronavirus (CoV), known as the 2019 novel coronavirus (2019-nCoV), resulted in a contagious acute respiratory infectious disease in December 2019 in Wuhan, Hubei Province, China. Its alarmingly quick transmission to many countries across the world and a considerable percentage of morbidity and mortality made the World Health Organization recognize it as a pandemic on March 11, 2020. The perceived risk of infection has led many research groups to study COVID-19 from different aspects. In this literature review, the phylogenetics and taxonomy of COVID-19 coronavirus, epidemiology, and respiratory viruses similar to COVID-19 and their mode of action are documented in an approach to understand the behavior of the current virus. Moreover, we suggest targeting the receptors of SARS-CoV and SARS-CoV-2 such as ACE2 and other proteins including 3CLpro and PLpro for improving antiviral activity and immune response against COVID-19 disease. Additionally, since phytochemicals play an essential role in complementary therapies for viral infections, we summarized different bioactive natural products against the mentioned respiratory viruses with a focus on influenza A, SARS-CoV, MERS, and COVID-19.Based on current literature, 130 compounds have antiviral potential, and of these, 94 metabolites demonstrated bioactivity against coronaviruses. Interestingly, these are classified in different groups of natural products, including alkaloids, flavonoids, terpenoids, and others. Most of these compounds comprise flavonoid skeletons. Based on our survey, xanthoangelol E (**88**), isolated from *Angelica keiskei* (Miq.) Koidz showed inhibitory activity against SARS-CoV PLpro with the best IC_50_ value of 1.2 μM. Additionally, hispidulin (**3**), quercetin (**6**), rutin (**8**), saikosaponin D (**36**), glycyrrhizin (**47**), and hesperetin (**55**) had remarkable antiviral potential against different viral infections. Among these compounds, quercetin (**6**) exhibited antiviral activities against influenza A, SARS-CoV, and COVID-19 and this seems to be a highly promising compound. In addition, our report discusses the obstacles and future perspectives to highlight the importance of developing screening programs to investigate potential natural medicines against COVID-19.

## Introduction

The emergence of viral pneumonia caused by a novel coronavirus (CoV), known as the 2019 novel coronavirus (2019-nCoV), resulted in a contagious acute respiratory infectious disease in December 2019 in Wuhan, Hubei Province, China (Zhu et al., 2020). Its alarmingly rapid transmission to many countries across the world and the high level of morbidity and mortality prompted the World Health Organization (WHO) to recognize it as a pandemic on March 11, 2020 (Hui et al., 2020). As of November 2, 2020, the number of total confirmed COVID-19 infections in the world is over 46 million, and the number of deaths is reportedly more than one million individuals. These numbers are continually increasing with no sign of respite (WHO Coronavirus Disease, 2020). Additionally, the WHO announced that the disease has led to disruptions in medical services, including many hospitals and clinics for the treatment of non-communicable diseases in countries throughout the world (WHO April 17th, 2020). Moreover, the global outbreak of the pandemic coronavirus has disturbed social, religious, economic, financial and political structures across the world (Mahar 2020).

SARS-CoV and MERS-CoV broke out in 2003 and 2012 respectively, and each resulted in approximately 800 deaths. SARS-CoV and MERS-CoV are both zoonotic pathogens originating from animals, and can cause life-threatening disease with potential to cause pandemics (Ksiazek et al., 2003; Zaki et al., 2012). Recent studies have proven that the SARS-CoV-2 shares 89.1% nucleotide similarity, and 79.5% sequence identity, to a group of SARS-like coronaviruses and it falls into the genus Betacoronavirus (Harapan et al., 2020; Wu et al., 2020; Zhou et al., 2020).

The insights provided in this study indicate that natural products (or secondary metabolites) isolated from plants and microorganisms with activity against respiratory viruses support further exploration of such sources for anti-COVID-19 agents. Additionally, our review focuses on the importance of respiratory viruses similar to COVID-19 and their mode of action. This serves not only to understand the mechanism of COVID-19, but also to promote the fundamental concept of structure-based drug design, which might assist in discovering promising anti-COVID-19 drugs.

## Epidemiology

Transmission routes, infection sources and susceptible hosts are three major conditions that influence transmission of infectious diseases. According to the latest statements of the National Health Commission of China, SARS-CoV-2 is transmitted to humans by respiratory droplets, close contact and contamination of surfaces, while transmission through aerosols is highly possible (Zhu et al., 2020).

Previous epidemics caused by SARS (2003) and MERS (2012) showed that these family members potentially cause widespread hospital outbreaks. Studies on SARS have shown that management of patients inside and outside the hospital is one of the most important steps in controlling the disease. For instance, the management and diagnosis of several imported cases to Vancouver (Canada) led to the rapid disease control and prevention of secondary transmission in the city. In return, unsuccessful control of the disease in Toronto (Canada) and Taipei (Taiwan) led to significant spread of the disease and hospitalization (Raoult et al., 2020).

The World Health Organization (WHO) declared SARS-CoV-2 as the sixth international public health concern and as a pandemic on March 11, 2020 (Zhang et al., 2020). Until now (November 2020) according to current laboratory tests, more than 46 million confirmed cases and one million deaths have been recorded around the world. According to the WHO report on 2 November, the most prevalent cases were recorded in America (20,807,415), Asia (13,626,009), Europe (10,324,515), Africa (1,796,748) and Oceania (including Australia, French Polynesia, Guam, New Zealand and Papua New Guinea) (41,916) (WHO Coronavirus Disease, 2020).

The mortality rate of SARS-CoV-2 has been reported in various studies and is estimated to be around 6.9% of confirmed cases but it may vary substantially (Zhu et al., 2020), for example ∼0.1% and ∼15.4% in Singapore and Belgium respectively (Johns Hopkins University). Accumulated results have revealed that the mortality rate of SARS-CoV-2 is lower than that of SARS-CoV or MERS-CoV, with rates of 10% and 37.1% respectively, but SARS-CoV-2 is ten times more contagious than the other viral infections (Bouadma et al., 2020). Statistical methods have determined that the infectious rate of SARS-CoV-2, which is presented as R0, is approximately between 1.3 and 6.5, with an average of 3.3 (Sen-Crowe et al., 2020).

Many reports indicate that SARS-CoV-2 has a long incubation period (between 2 and 14 days) with high asymptomatic transmission capability, and this feature may well be a contributing factor to the rapid spreading of the virus (Ahn et al., 2020). The pattern of the outbreak shows that all races and ages are susceptible to the infection. However, the rates of severe disease and mortality are more prevalent in the elderly and individuals with underlying disease such as cardiovascular diseases, diabetes and high blood pressure (Chen et al., 2020).

## Phylogeny and Taxonomy of COVID-19 Coronavirus

The latest classification divides the Coronaviridae family into two subfamilies, the Coronavirinae and the Letovirinae (García 2020; Scagliarini and Alberti, 2020). There are four genera in the Coronavirinae subfamily including Alpha, Beta, Gamma and Delta-coronavirus. Thus far, seven human pathogenic coronaviruses have been identified, with the NL63 and 229 E in the Alpha genus, and OC43, HKU1, SARS, MERS and SARS-CoV-2 belonging to the Beta genus (Wang et al., 2020).

SARS-CoV-2 is an enveloped RNA virus possessing a positive sense, single-stranded genome around 29.9 Kb. A large part (about two-thirds) at the 5′ end of viral genome encodes the pp1ab and pp1a polyproteins, fractured into 15 non-structural proteins which consist of nsp1-16. On the opposite side (3′ end), the virus genome encodes four structural proteins including surface spike (S), matrix (M), envelope (E), nucleocapsid (N) and also eight accessory proteins. Structural and accessory proteins participate in virus morphogenesis and immune system interference, while non-structural proteins are involved in virus replication. The spike glycoprotein is necessary for the attachment to the host cell receptors and determines tissue tropism (Zhang et al., 2020).

Analysis of phylogenetic results has shown that SARS-CoV-2 is genetically related to bat-SL-CoVZC45 and bat-SL-CoVZXC21 (89% identity), but has less similarity to SARS-CoV (79%) and MERS-CoV (50%) (Lai et al., 2020). In this regard, analysis of protein sequences showed that SARS-CoV-2 was evolutionarily most related to SARS-CoV. Evaluation of structural proteins indicated that the envelope, nucleocapsid and spike protein have 96%, 89.6%, and 77% sequence identity, respectively, compared to SARS-CoV (Lan et al., 2020).

Other research has shown SARS-CoV-2 to have the highest level of genetic similarity with Malaya pangolin coronaviruses such as *Pan*_SL-CoV_GD (91.2%) and *Pan*_SL-CoV_GX (85.4%). As an intermediate host, it seems that pangolins may have played an important role in transmitting the virus to humans (Forster et al., 2020).

In a recent study by Forster et al. (Forster et al., 2020) genetic characterization of 160 positive samples of SARS-CoV-2 were evaluated from across the world. Phylogenetic analysis showed that three variants (A, B, C) are available according to amino acid changes. Variant A is ancestral type and exists with C variant in European and American patients. The B variant is most common in Asian patients (Forster et al., 2020).

## Respiratory Viruses Similar to COVID-19 and Their Mode of Action

Viral respiratory infections cause life-threatening disease in many people worldwide and affect the lives of millions of people worldwide each year. Global interest in respiratory viruses has recently increased substantially owing to the emergence of some new viruses including SARS-CoV, avian influenza A H5N1, H7N9, H1N1v2009, and Mers-CoV (Visseaux et al., 2017). This review will therefore exclusively focus on influenza viruses and coronaviruses, both of which are known to cause respiratory infections.

### Influenza Virus

Influenza virus belongs to the Orthomyxoviridae family, which contains three genera of influenza viruses, namely influenza A, B and C viruses. These are classified according to antigenic differences between their nucleoprotein (NP) and matrix 1 (M1) proteins. Types A and B are responsible for seasonal flu epidemics each year. Influenza virus infection remains one of the most serious threats to human health and causes epidemics or pandemics. Influenza viruses are responsible for acute contagious respiratory infections (Memoli et al., 2008). Influenza A viruses cause the most virulent disease among the three influenza types and, based on the antibody responses to these viruses, they may be divided into different serotypes (Zowalaty et al., 2011). Influenza A viruses contain seven or eight pieces of single-stranded, segmented negative-sense RNA. The genomes of influenza A viruses encode eleven proteins including HA, NA, NP, PB1, PB1-F2, PB2, NS1, NEP, PA, M1, and M2. The main antigenic factors of influenza A and B viruses are the hemagglutinin (HA) and neuraminidase (NA) *trans*-membrane glycoprotein knobs (Kampus et al., 2006). The A virus can be divided into subtypes based on the antigenic nature of their HA (16 subtypes) and NA (9 subtypes) glycoproteins (Klenk et al., 2008).

HA is the main influenza virus antigen and its antigenic sites (A, B, C, D, and E) are presented at the head of the molecule, while the feet are fixed in the viral membrane. HA influences the extent of infection into the host organism. The HA protein attaches to sialic acid-containing glycoprotein and glycolipid receptors on the cell surface (Kollerova and Betakova 2006). NA (also called sialidase) releases progeny virions from infected cells by cleaving sialic acid from the HA molecule, from other NA molecules and from receptors at the cell surface. NA may promote the movement of viruses through respiratory tract mucus, in this way enhancing viral infectivity. When the influenza virus is deficient in NA activity, this results in aggregation of virus progeny at the surface of the infected cell, which will severely impair further spread of viruses to other cells (Zhang et al., 2006). Also, NA mutations can result in the ability of influenza A viruses to adapt to novel environments, enabling the virus to jump host species barriers (Suzuki 2005). These two proteins are targets for antiviral drugs (Wilson and Von 2003) and are capable of exciting subtype-specific immune responses. M1 and M2 are matrix proteins. The M1 protein coats inside the viral envelope and has several regulatory actions, performed by interaction with host cell components. M2 is a proton-selective ion channel which only passes the proton ions. It causes hydrogen ions to enter the viral particle, lowering the pH inside of the virus and causing dissociation of M1 from RNP, finally uncoating the virus. PA encodes an RNA polymerase. PB1 encodes an RNA polymerase, while PB1-F2 protein induces apoptosis using different reading frames from the same RNA segment and PB2 encodes an RNA polymerase (Kampus et al., 2006).

The currently-approved classes of small molecule drugs for treating influenza viral infections include neuraminidase inhibitors (zanamivir and oseltamivir), as well as M2 channel blockers (amantadine and rimantadine), and viral polymerase inhibitors (favipiravir and baloxavir marboxi) (Lagoja and De Clercq 2008; Furuta et al., 2017). The efficacy of favipiravir in influenza treatment has lately been questioned owing to a lack of efficacy in primary human airway cells (Yoon et al., 2018). In 2010, two new neuraminidase inhibitors (NAIs) known as peramivir and laninamivir were licensed in Japan (Shetty and Peek 2012).

Studies have also shown that influenza infections result in the uncontrolled increase of pro-inflammatory cytokines, which makes this infection a strong risk factor for severe complications which may be terminal (De Jong et al., 2006; Rothberg and Haessler 2010). Influenza infection can induce a cytokine storm. The cytokine storm or ‘hypercytokinemia’ is a systemic expression of a healthy and strong immune system, and is potentially lethal, consisting of positive feedback between cytokines and immune cells with high levels of various cytokines (Osterholm 2005). These cytokines activate guanosine triphosphatase (GTPase) proteins by isoprenylation, which adds the isoprenyl groups such as farnesyl pyrophosphate (FPP) or geranylgeranyl pyrophosphate (GGPP) to the GTPase proteins. This causes Nuclear Factor Kappa Beta (NF-κB) movement to the nucleus, recruiting Toll Like Receptor-7 (TLR-7) in the endosomal compartment which recognizes ssRNA (Kawai and Akira 2006) to express the target genes for cytokines such as TNFα/β, IL-1α/β, IFNα/γ, IL-6, MIP-1α and IL-8. This in turn enables the attraction of different immune effectors and finally leads to inflammation (Osterholm 2005; Piqueras et al., 2006). The assembly of prostaglandins, especially E2, triggers complex thermoregulatory mechanisms to increase the temperature of the body (Brydon et al., 2005). Some anti-inflammatory drugs can block or decrease influenza virus infection by a reduction in protein isoprenylation in membrane structures and continuing pathways (Blanco-Colio et al., 2003). Some effective alternative therapeutics to vaccines and conventional antiviral drugs can be based on anti-inflammatory and immunomodulatory agents (Fedson 2006).

### Coronaviruses

The classification of CoVs has been based on genomic organization, similarities in the genomic sequence, replication strategies, antigenic properties of viral proteins, as well as structural characteristics of virions, cytopathogenic, pathogenic and physicochemical properties (Lai and Cavanagh, 1997). Four subgroups of CoVs (Alpha, Beta, Gamma, and Delta) contain pathogens of veterinary or human importance. Common cold symptoms in immunocompetent individuals are caused by AlphaCoVs and the BetaCoVs (HCoV-OC43 and HKU1) (Chiu et al., 2005; Jean et al., 2013). The unique mechanism of viral recombination and an inherently high mutation rate combined with a high frequency of recombination, results in the diversity of hosts and genomic features amongst CoVs (Lai and Cavanagh 1997). A different genus of CoVs can infect birds, whales, swine, cats, dogs, bats, rodents and humans (Abolnik, 2015).

There are four main structural proteins in genomes of coronaviruses including the Spike (S), Membrane (M), Envelope (E) glycoproteins, and Nucleocapsid (N) protein. Besides these structural proteins, different CoVs encode special structural and accessory proteins, such as Hemagglutinin Esterase (HE) protein, 3a/b protein, and 4a/b protein. HE can only be found in some Beta coronaviruses but envelope proteins and nucleocapsid protein are present in all virions (Lissenberg et al., 2005).

S proteins, located outside the virion, bind to the virion membrane via the C-terminal transmembrane regions and they also interact with M proteins (Consortium, 2004). Also, the attachment of virions to surface receptors in the plasma membranes of host cells can happen through the N-terminus of the S proteins (Lewicki and Gallagher, 2002). Additionally, the S proteins form homotrimers, which allow sun-like morphologies to form, giving the name to coronaviruses (Bárcena et al., 2009).

The M protein forms a complex in the absence of the S protein (Vennema et al., 1996). Glycosylation of M proteins in the Golgi apparatus is vital for the virion to fuse into the cell and to make protein antigenic (de Haan et al., 2003). By binding genomic RNA, the N protein forms a complex and the formation of interacting virions in this endoplasmic reticulum-Golgi apparatus intermediate compartment (ERGIC) with this complex is triggered by the M protein (Narayanan and Makino, 2001).

E Glycoproteins are small transmembrane proteins which play an essential role in the assembly and morphogenesis of virions within the cell. Also, the N-terminus of the E proteins allows attachment to the membrane of viruses. E Glycoproteins and the membrane glycoprotein (M) are expressed together with mammalian expression vectors to form virus-like structures within the cell (Baudoux et al., 1998).

N proteins, with a flexible structure, are capable of binding to a helix. They are localized in both the replication/transcriptional region of the coronaviruses and the ERGIC region where the virus is collected so it plays an essential role in virion structure, replication and transcription of coronaviruses (Stertz et al., 2007).

Coronavirus infection initiates following recognition of a specific receptor on the host cell surface by the coronaviral S protein and subsequent internalization of the virion core, which occurs either by direct fusion of the viral membrane with the plasma membrane or via endocytosis (Blau and Holmes, 2001). Some species (eg, SARS and CoV) use the N-terminus, while others use the C-terminus of the S1 site of the receptor-binding domains (RBD) (Islam et al., 2020). Attaching to specific cellular receptors triggers a conformational change in the spike which subsequently mediates fusion between the viral and cell membranes, causing release of the nucleocapsid into the cell (Brian and Baric, 2005). The trimeric S protein is cleaved into an amino (N)-terminal S1 subunit and a carboxyl (C)-terminal S2 subunit (Belouzard et al., 2012). The S1 subunit contains a receptor-binding domain (RBD), which binds the cellular receptor angiotensin-converting enzyme 2 (ACE2), while S2 is responsible for membrane fusion (Li et al., 2005a; Li et al. 2005b; Li et al. 2005c). Different host cell receptors are recognized by different coronaviruses such as Heparan Sulfate Proteoglycans, ACE2, Aminopeptidase N, Heat Shock Protein A5 (HSPA5), furin, and O-Acetylated Sialic Acid (Belouzard et al., 2012; Huang et al., 2015; Hasan et al., 2020; Ibrahim et al., 2020). ACE2 is an analogue of the angiotensin converting enzyme type I (ACE) and part of the renin–angiotensin system responsible for blood pressure regulation (da Silva Antonio et al., 2020). Some analysis suggested that SARS-CoV-2 recognizes human ACE2 more efficiently than SARS-CoV, increasing the capacity of SARS-CoV-2 to transmit from human to human (Wan et al., 2020). Different studies have proven that the affinity between the viral RBD and host ACE2 in the initial SARS-CoV attachment step can determine if a host is susceptible to SARS-CoV infection (Li et al., 2004; Li et al., 2005a; Li et al., 2005b; Li et al., 2005c; McCray et al., 2007). In 2007, it was demonstrated that overexpression of human ACE2 caused an enhancement in disease severity in a mouse model of SARS-CoV infection, showing that ACE2 is essential for viral entry into the cell (Yang et al., 2007). ACE2 also functions to protect against lung injury, thus, because the virus deregulates the lung protective pathway, SARS-CoV has the potential to become highly lethal (Kuba et al., 2005). ACE2 acts as the entry receptor, and the host enzyme transmembrane protease serine 2 (TMPRSS2) is essential for S protein priming. This enzyme facilitates viral particle entry into the host cells, and its inhibition blocks virus fusion with ACE2 (Cheng et al., 2020a; Cheng et al., 2020b). After entering the cytoplasm, the virus particle releases a single-stranded, non-segmented RNA virus with the largest known RNA genome (gRNA) (Wege et al., 1978). The genome comprises seven genes. These are organized into 5’ non-structural protein-coding regions containing the replicase genes (gene 1), comprising two-thirds of the genome, and 3’ structural and nonessential accessory protein coding regions which in turn comprises the genes 2–7 (Weiss and Navas-Martin, 2005). Two large open reading frames, ORF1a and 1b, which translate into two large polypeptides (pp1a and pp1b), are encoded by the replicase gene 1. Pp1a and pp1b mediate all the functions necessary for viral replication and transcription (Thiel et al., 2001). Sixteen non-structural proteins (nsp) are converted with the contribution of pp1a and pp1b (Baranov et al., 2005; Tok and Tatar 2017). A Double-Membrane Vesicle (DMV), which is a virus Replication and Transcription Complex (RTC), is formed by these nsp proteins. The role of these 16 proteins, especially nsp3, in the virion structure, as well as the replication and transcription of CoV is notable (Denison 2008; Tok and Tatar 2017). Also, the major viral structural proteins and the accessory proteins are encoded by genes 2 to 7. The accessory proteins have been proven to be essential for virus-cell receptor binding (Goldsmith et al., 2004; Stertz et al., 2007).

All coronaviruses encode one main proteinase. This main proteinase is commonly referred to as ‘3C-like’. Thus, the coronavirus enzyme is called coronavirus 3C-like proteinase, or 3CLpro. The 3CLpro is analogous to the main picornaviral protease 3Cpro. Coronaviruses also encode one (group 3) or two (groups 1 and 2) papain-like proteases, termed PLP1pro and PLP2p (Ziebuhr et al., 2000). Upon transcription of the genome, a polypeptide is produced by BetaCoVs. The proteolytic cleavage of the polypeptide results in the generation of various proteins. Proteolytic processing is mediated by papain-like protease (PLpro) and 3-chymotrypsin-like protease (3CLpro). The protease 3CLpro cleaves the polyprotein at 11 distinct sites to result in various non-structural proteins critical for viral replication. Thus, the role of 3CLpro in the replication is substantial. The 3CLpro is located at the 3’ end, which is characterized by excessive variability (Anand et al., 2003). The papain-like protease (PLpro) is an important cysteine protease in SARS-CoV virus replication. It cleaves ubiquitin chains and affects deISGylation and is responsible for processing the viral polyprotein, as well as processing the viral polypeptide into functional proteins (Lenschow et al., 2007).

In the next section, we are going to discuss natural products with efficacy against some HCoVs, including SARS-CoV, MERS-CoV, and SARS-CoV-2, which belong to BetaCoVs. The genomic organization of BetaCoVs consists of a 5′-untranslated region (UTR), a replicase complex (orf1ab), encoding non-structural proteins (nsps), S protein gene, E protein gene, an M protein gene, an N protein gene, 3’-UTR, as well as several unidentified non-structural open reading frames (Zhu et al., 2020).

The viral main proteinase (3CLpro) controls the activities of the coronavirus replication complex. It can be an attractive target for therapy. Also, due to the key role of PLpro it can be considered as an important target for antiviral agents (Anand et al., 2003). ACE2 is a functional receptor for SARS coronavirus (SARS-CoV) and SARS-CoV-2 to enter the host target cells (Hoffmann et al., 2020), and thus ACE2 inhibition can be considered for development of antiviral agents against SARS-CoV and SARS-CoV-2. These three proteins therefore provide attractive targets for drug development.

## Natural Products Against Respiratory Viruses

The failure of many conventional drugs against viral infections combined with the onset of specific viral resistance has led to an increasing interest in plants as promising antiviral agents (Lin et al., 2014). Nature provides an immense library of novel chemicals to explore for the development of drugs to treat various ailments including viral diseases (Denaro et al., 2020). Since natural compounds, including phenolic acids, terpenes, flavonoids, coumarins, lignans, alkaloids and proteins play an essential role in inhibiting viruses and acting as complementary therapies against viral infections (Daglia 2012), we reviewed active natural products against respiratory viruses with a focus on influenza A, SARS-CoV, MERS, and SARS-CoV-2, the cause of the current pandemic of COVID-19.

### Natural Products Against Influenza Virus

Resistance to some drugs for the treatment of influenza viral infections is an ongoing problem that has been reported in several cases (Baz et al., 2009; Moscona 2009) and the efficacy of favipiravir in influenza treatment has been doubted because of a lack of efficacy in primary human airway cells (Yoon et al., 2018). Therefore, there exists a significant unmet medical need for novel effective antiviral drugs to combat this disease, and natural products can be considered as an important source of antiviral drugs against influenza.

Although favipiravir, oseltamivir, zanamivir, and peramivir are synthetic compounds, they are inspired by nature with the original active compound of natural origin (Langeder et al., 2020). Additionally, natural-based agents are used most frequently for the treatment of acute respiratory tract infection, especially in children, because of the lack of specific antiviral drugs, easy access and low cost (Lucas et al., 2018). This section provides an overview of natural products affecting the influenza A virus.

Flavonoids are phenolic substances which are one of the most numerous and widespread groups of natural constituents. There are ten major sub-groups of flavonoids, i.e. aurones, biflavonoids, catechins, chalcones, flavanones, flavanonols, flavans, flavones, flavonols and isoflavones (Harborne et al., 2013). Some flavonoids are reported to have antiviral effects against different types of influenza viruses. In 2018 three flavonoids including 6-hydroxyluteolin 7-O-β-d-glucoside, nepitrin (**1**) and homoplantaginin were isolated from the methanol extract of *Salvia plebeia* R. Br. These compounds were found to be active against influenza virus H1N1A/PR/9/34 neuraminidase (Bang et al., 2018). Matteflavoside G (**2**), a flavonoid isolated from the rhizomes of *Matteuccia struthiopteris* (L.) Tod (currently accepted name Onoclea struthiopteris Roth), showed significant inhibitory activity against the H1N1 influenza virus neuraminidase with an EC50 value of 6.8 ± 1.1 μM and an SI value of 34.4 (Li et al., 2015). In 2016, three flavonoids (**3**–**5**) were isolated from the methanol extract of aerial part of *Salvia plebeia* R. Br. These compounds showed activity against H1N1 neuraminidase in dose-dependent manners with IC_50_ values ranging from 11.18 ± 1.73 to 19.83 ± 2.28 μM. Also, compounds 3 and 4 reduced cytopathic effects of the H1N1 virus during replication (Bang et al., 2016). Flavonoids including quercetin (**6**), isoquercetin (**7**), and rutin (**8**), isolated from the methanol extract of *Capparis sinaica* Veill (accepted scientific name Capparis spinosa var. aegyptia (Lam.) Boiss.), demonstrated a reduction in the virus titer by 68.13%, 79.66% and 73.22% inhibition at a concentration of 1 ng/ml, respectively (Ibrahim et al., 2013). Quercetin (**6**) and rutin (**8**) are reported to inhibit the viral neuraminidase activities *in vitro* and the influenza infection in animal models (Savov et al., 2006; Liu et al., 2008). Quercetin (**6**) and rutin (**8**) were isolated from the hydroalcoholic extract of *Humulus lupulus* L. This extract was found to inhibit replication of various viral strains, at a different time from infection (Di Sotto et al., 2018). Moreover, Mehrbod et al. studied *in vitro* anti-influenza virus a potential of extracts of five South African medicinal plants, including *Clerodendrum glabrum* E. Mey (current name Volkameria glabra (E.Mey.) Mabb. & Y.W.Yuan)*, Cussonia spicata* Thunb.*, Rapanea melanophloeos* (L.) Mez (current name *Myrsine melanophloeos* (L.) R. Br. ex Sweet)*, Pittosporum viridiflorum* Sims and *Tabernaemontana ventricosa* Hochst. ex A. DC., species which are known in traditional medicine to manage several diseases such as inflammatory and respiratory diseases (Mehrbod et al., 2018). The study indicated that in all types of combined treatments such as pre- and post-penetration combined treatments, the methanol leaf extracts of *Rapanea melanophloeos* had an EC50 value of 113.3 μg/ml and the methanol, 100% and 30% ethanol and acetone leaf extracts of *P. viridiflorum* had EC50 values of 3.6, 3.4, 19.2, and 82.3 μg/ml, respectively, with highly significant effects against viral titer (*p* ≤ 0.01).

Additionally, the authors found that quercetin-3-o-α-l-rhamnopyranoside (**9**) isolated from *R. melanophloeos* inhibited the viral titre by 6 logs (*p* < 0.01) in the simultaneous procedure at a concentration of 150 μg/ml (Mehrbod et al., 2018). Also, further experiments on quercetin-3-o-α-l-rhamnopyranoside (**9**) showed that the compound in combination with H1N1 was able to induce apoptosis and showed an immunomodulatory effect on some selected pro- and anti-inflammatory cytokines (Mehrbod et al., 2019) (−)-Epigallocatechin-3-gallate (EGCG) (**10**) is one of the major flavonoid components of green tea. Kim and co-workers proved that it damaged the viral membrane and blocked viral penetration into cells and marginally suppressed the viral and nonviral neuraminidase (NA) activity in an enzyme-based assay system (Kim et al., 2013). The association of influenza A virus infections with secondary complications caused by bacterial pathogens, mostly *Streptococcus pneumoniae,* has been reported (McCullers 2014). Two prenylated flavonoid derivatives named sanggenon G (**11**) and sanggenol A (**12**) were found to act as dual inhibitors of both viral and bacterial NAs. Interestingly in contrast to the approved NA inhibitor oseltamivir, these compounds inhibited planktonic growth and also biofilm formation of pneumococci (Grienke et al., 2016).

According to the results (E)-4, 2′, 4′-trihydroxy-6′-methoxy-3′,5′-dimethylchalcone (**13**) and 2.2′,4′-trihydroxy-6′- methoxy-3′,5′-dimethylchalcone (**14**) possessed the strongest effects against NA (Dao et al., 2010). In the screening of natural products for anti-influenza potential, two chalcones named echinantin (**15**) and isoliquiritigenin (**16**) revealed strong inhibitory action against various neuraminidases originating from the influenza viral strains H1N1, H9N2, novel H1N1 (WT), and oseltamivir-resistant novel H1N1 (H274Y) expressed in 293T cells. Echinantin was identified as the most active compound against NA derived from the novel H1N1 influenza with an IC_50_ of 2.49 ± 0.14 μg/ml. Additionally, the efficacy of oseltamivir was increased against H274Y neuraminidase in the presence of compound **15** (5 L µM) (Dao et al., 2011). Also, isoliquiritigenin (**16**) was among eighteen polyphenols isolated from methanol extracts of the roots of *Glycyrrhiza uralensis* Fisch. ex DC. with neuraminidase inhibitory activity (Ryu et al., 2010). Some biflavonoids like ginkgetin (**17**), hinokiflavone (**18**), and 4′-*O*-methylochnaflavone (**19**) were successfully identified as potential NA inhibitors (Miki et al., 2007; Mercader and Pomilio 2010; Kirchmair et al., 2011). Fourteen C-methylated flavonoids including chalcones, flavanones, isoflavones, and one flavanonol were isolated from the methanol extract of *Cleistocalyx operculatus* (Roxb.) Merr. and L.M.Perry (current name Syzygium nervosum A.Cunn. ex DC.).

Coumarins are a very large family of natural products. Many coumarin derivatives have been isolated from plants (Cao et al., 2019). Some coumarins have been reported to possess antiviral effects against influenza A virus. Glycyrol (**20**) is a coumarin isolated from *G. uralensis* with a strong inhibitory effect (IC_50_ = 3.1 μM) (Ryu et al., 2010). Bioassay-guided fractionation of the extract of *Ferula assa-foetida* L (also known as Ferula foetida (Bunge) Regel) afforded twenty sesquiterpene coumarins. Among the isolated compounds, nine compounds were more potent against influenza A virus (H1N1) with IC_50_ between 0.26–0.86 μg/ml than amantadine with an IC_50_ of 0.92 μg/ml. Between all isolated sesquiterpene coumarins, methyl galbanate (**21**) was the most potent (IC_50_ = 0.26 μg/ml) (Lee et al., 2009).

Ten xanthone derivatives were isolated from the ethyl acetate-soluble extract of *Polygala karensium* Kurz. The inhibitory effect of five compounds (**22**–**26**) with a hydroxy group at C-1 against neuraminidases from various influenza viral strains, notably H1N1, H9N2, novel H1N1 and oseltamivir-resistant novel H1N1, was reported. Moreover, the cytopathic potential of H1N1 swine influenza virus in MDCK cells was reduced by these compounds (Dao et al., 2012).

Rosmarinic acid methyl ester (methyl rosmarinate) (**27**) isolated from the methanol extract of erial parts of *Salvia plebeia* R. Br was active against H1N1 neuraminidase with an IC_50_ value of 16.65 ± 0.91 µM. Also, this compound reduced cytopathic effects of the H1N1 virus during replication (Bang et al., 2016). Inotilone (**28**) and 4-(3, 4-dihydroxyphenyl)-3-buten-2-one (**29**), isolated from the mushroom *Phellinus linteus*, were effective against H1N1 neuraminidase and the influenza A/WS/33 virus. They had neuraminidase inhibitory activity with IC_50_ values of 29.1 and 125.6 µM, respectively (Hwang et al., 2014).

Embeline (**30**) is an alkyl-benzoquinone isolated from the ethyl acetate extract of fruits of *Embelia ribes* Burm. f.. This compound demonstrated antiviral activity against the H1N1 influenza virus, with an IC_50_ of 0.3 µM and an SI of 10, which provides support for further research on this molecule. It was proved that embelin (**30**) was most effective when added at the early stages of the viral life cycle (Hossan et al., 2018).

Four diarylheptanoids out of six isolated constituents from the ethanol extract of the seed of *Alpinia katsumadai* K. Schum (current name Alpinia hainanensis K.Schum.). exhibited inhibitory activities *in vitro* at low micromolar levels against human influenza virus A/PR/8/34 of subtype H1N1. Katsumadain A (**31**) was the most potent compound (IC_50_ = 1.05 ± 0.42 μM) and the most active inhibitor of the NA of four H1N1 swine influenza viruses, with IC_50_ values from 0.9 to 1.64 μM, and also had antiviral effects in plaque reduction assays (Grienke et al., 2010).

Chang et al. (Chang et al., 2016) screened a library of natural components and identified a novel anti-influenza compound with broad-spectrum activity against seasonal influenza A and B viruses. The compound 1,3,4,6-tetra-O-galloyl-b-d-glucopyranoside (TGBG) (**32**) obtained from *Euphorbia humifusa* Willd. was the most active compound against two seasonal influenza A strains, A/California/07/2009 (H1N1) and A/Perth/16/2009 (H3N2), as well as seasonal influenza B strain B/Florida/04/2006. It was proven that the mode of action of TGBG (**32**) was different from the FDA-approved anti-influenza drugs. It significantly inhibited the nuclear export of influenza nucleoproteins (NP) and suppressed the Akt signaling pathway in a dose-dependent manner (Chang et al., 2016).

In 2017, cynanversicoside A (**33**) was isolated from the ethyl acetate extract of *Cynanchum paniculatum* (Bunge) Kitag. ex H. Hara (current name *Vincetoxicum mukdenense* Kitag.). It showed potent anti-inflammatory and antiviral effects on influenza A virus -infected MPMEC by the regulation of NF-κB and MAPK signaling pathways (Wei et al., 2017). Forsythoside A (**34**) was isolated from the methanol extract of *Forsythia suspensa* (Thunb.) Vahl fruit. It increased the survival rate of infected mice in an influenza virus infection model and reduced the viral titers of different influenza virus subtypes in cell cultures. Forsythoside A (**34**) caused a reduction in the influenza M1 protein, which limited the virus spread (Law et al., 2017).

In 2016, the influenza virus NA inhibitory activities of some naturally occurring chlorogenic acids against NAs from *Clostridium perfringens*, H5N1, and recombinant H5N1 (N-His)-Tag were investigated. According to the findings, all chlorogenic acids isolated from *C. perfringens* and selected derivatives demonstrated considerable activities against NAs (Karar et al., 2016).

It is beneficial for a plant-based natural antiviral remedy to have multiple useful activities such as treating secondary infections as well as symptoms associated with influenza and other viral infections.

**FIGURE 1:**
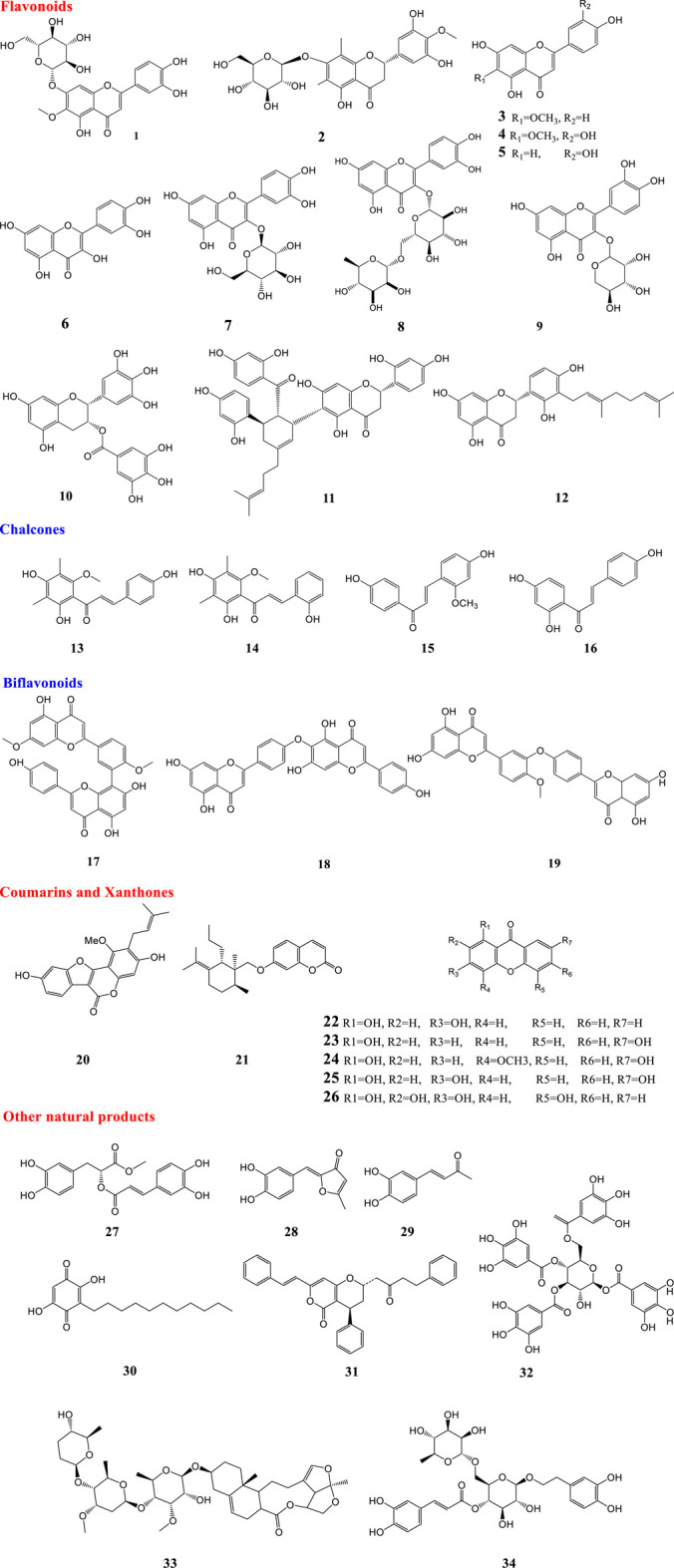
Structures of natural products against influenza virus.

*Fructus Gardeniae*, the dry ripe fruits of *Gardenia jasminoides* J. Ellis, is widely used as a traditional medicine in several Asian countries. Bioassay-guided fractionation of the methanol extract from *Fructus Gardeniae* led to the discovery of bioactive natural products with antiviral potential against influenza virus strain A/FM/1/47-MA. The target fraction was administered orally to rats, blood was collected, and the compounds in rat serum after oral administration were separated and characterized. Thirteen compounds including iridoid glycosides, phenylpropanoids, and their derivatives were confirmed or tentatively identified (Yang et al., 2012). The characteristics of natural products against influenza virus have been summarized in Table 1. Structures of natural products against influenza virus are depicted in Figure 1.

**TABLE 1:** Characteristics of some natural products against influenza virus.

Influenza subtype						Plant/Solvent					Compounds						Effect				Ref			
H1N1/PR8						*Salvia plebeia* R.Br*/*methanol					6-Hydroxyluteolin 7-O-β-d-glucoside, Nepitrin (**1**) and homoplantaginin						NA inhibitor				Bang et al. (2018)			
H1N1/PR8						*Matteuccia struthiopteris* (L.) Tod. (Onoclea struthiopteris Roth)*/*ethanol					Matteflavoside G (**2**)						NA inhibitor				Li et al. (2015)			
H1N1/PR8						*Salvia plebeian* R.Br*/*methanol					Hispidulin (**3**), Nepetin (**4**), Methyl ester (**5**)						NA inhibitor				Bang et al. (2016)		
H5N1						*Capparis sinaica* Veill. (Capparis spinosa var. aegyptia (Lam.) Boiss.) */*methanol					Quercetin **(6)**, Isoquercetin (**7**), Rutin (**8**)						NA inhibitor				Ibrahim et al. (2013)		
H1N1/PR8 H1N1/NWS H7N1						*Humulus lupulus* L*./*hydroalcoholic					Quercetin (**6**), Rutin (**8**)						Interference with redox-sensitive pathways				Di Sotto et al. (2018)			
H1N1/PR8						*Rapanea melanophloeos* (L.) Mez (*Myrsine melanophloeos* (L.) R.Br. ex Sweet) and *Pittosporum viridiflorum* Sims */*methanol					Crude						HA inhibitor				Mehrbod et al. (2018)			
H1N1/PR8						*Rapanea melanophloeos* (L.) Mez *(Myrsine melanophloeos* (L.) R.Br. ex Sweet)*/*methanol					Quercetin-3-o-α-l-rhamnopyranoside (**9**)						Blocking the virus receptor				Mehrbod et al. (2018)		
H1N1/PR8						*Rapanea melanophloeos* (L.) Mez *(Myrsine melanophloeos* (L.) R.Br. ex Sweet)*/*methanol					Quercetin-3-o-α-l-rhamnopyranoside (**9**)						Interaction with M2, NA and RhoA				Mehrbod et al., (2019)					
H1N1/PR8, H3N2						green tea/ND					Epigallocatechin-3-gallate **(10)**						NA inhibitor				Kim et al. (2013)		
H1N1						White mulberry root*/*methanol					Sanggenon G **(11)**, Sanggenol A **(12)**						NA inhibitor				Grienke et al. (2018)		
H1N1, H9N2						*Cleistocalyx operculatus* (Roxb.) Merr. & L.M.Perry (Syzygium nervosum A.Cunn. ex DC.) */*methanol					(E)-4, 2′, 4′-trihydroxy6′-methoxy-3′,5′-dimethylchalcone (**13**), 2.2′,4′-trihydroxy-6′- methoxy-3′,5′-dimethylchalcone **(14)**						NA inhibitor				Dao et al. (2010)		
H1N1, H9N2, H1N1 (H274Y4Y)						*Glycyrrhiza inflata* Batalin*/*acetone					Echinantin **(15)**, Isoliquiritigenin **(16)**						NA inhibitor				Dao et al. (2011)		
H1N1/PR8 H3N2						*Ginkgo biloba* L.*/*ND					Ginkgetin (**17**), Hinokiflavone (**18**), 4′-O-methylochnaflavone (**19**)						NA inhibitor				Miki et al. (2007)		
H1N1						*Glycyrrhiza uralensis* Fisch. ex DC.*/*methanol					Isoliquiritigenin, glycyrol **(20)**						NA inhibitor				Ryu et al. (2010)		
H1N1						*Ferula assa-foetida* L. (Ferula foetida (Bunge) Regel)*/*methanol					Methyl galbanate **(21)**						ND				Lee et al. (2009)		
H1N1, H9N2						*Polygala karensium* Kurz*/*ethyl acetate					1,7-Dihydroxyxanthone, 1,7-dihydroxy-4-methoxyxanthone, 1,3,7-trihydroxyxanthone, 1.2,3,5-tetrahydroxyxanthone **(22–26)**						NA inhibitors				Dao et al. (2012)		
H1N1/PR8						*Salvia plebeian* R.Br*/*methanol					Rosmarinic acid methyl ester (**27**)						NA inhibitor				Bang et al. (2016)		
H1N1						*Phellinus linteus/*ethyl acetate					Inotilone **(28)**, 4-(3, 4-dihydroxyphenyl)-3-buten-2-one **(29)**						NA inhibitor				Hwang et al. (2014)		
H1N1,H5N2, H3N2						*Embelia ribes* Burm.f.*/*ethyl acetate					Embeline **(30)**						HA inhibitor				Hossan et al. (2018)		
H1N1						*Alpinia katsumadai* K.Schum. (Alpinia hainanensis K.Schum.) */*ethanol					Katsumadain A **(31)**						NA inhibitor				Grienke et al. (2010)		
H1N1, H3N2, flu B						*Euphorbia humifusa* Willd.*/*ND					1,3,4,6-tetra-O-galloyl-b-d-glucopyranoside **(32)**						Inhibits the nuclear export of NP				Chang et al. (2016)	
H1N1						*Cynanchum paniculatum* (Bunge) Kitag. ex H.Hara (*Vincetoxicum mukdenense* Kitag.)*/*ethyl acetate					Cynanversicoside A **(33)**						Suppressing NF-κB and MAPKs activation				Wei et al. (2017)	
H5N1						*Sonchus oleraceus* L. and *Armeria maritima* (Mill.) Willd.*/*Methanol					Chlorogenic acid derivatives						NA inhibitor				Karar et al. (2016)	
H1N1, H9N2, H3N2						*Forsythia suspense* (Thunb.) Vahl */*Methanol					Forsythoside A **(34)**						Reduction in the M1 protein				Law et al. (2017)	
H1N1						*Gardenia jasminoides* J.Ellis/methanol					Iridoids glycosides and phenylpropanoid derivatives						ND				Yang et al. (2012)			

NA, neuraminidase; ND, not-determined; NP, nuclear protein; HA, hemagglutinin; M1, matrix protein; RhoA, Ras homolog family member A; MAPKs, Mitogen-activated protein kinases.

### Natural Products Against Coronaviruses

Human coronaviruses are responsible for worldwide epidemic outbreaks and infections, and their rapid spread globally is of major concern. SARS-CoV, MERS-CoV and SARS-CoV-2 are considered as the most emergent CoVs. Although several drugs, such as ribavirin, lopinavir-ritonavir, interferon, and corticosteroids, have been used as treatment with some efficacy in patients infected with SARS-CoV or MERS-CoV (Shalhoub et al., 2015; Shen et al., 2019), no specific treatments or vaccines are available against HCoVs.

In the absence of effective treatments for HCoV infections, natural products might be potential alternative therapies. Many natural products have been tested in terms of their activity against coronaviruses and showed much potential in coronavirus treatment (Adem et al., 2020). Bioactive compounds use different mechanisms to inhibit coronaviruses, and inhibition of ACE2, 3CLpro, and PLpro are most common. Here we mention some of these natural products which have been proven to be active against coronaviruses.

**FIGURE 2:**
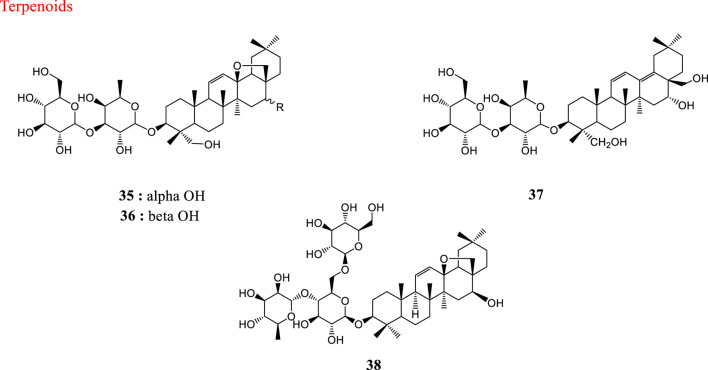
Structures of natural products against HCoV-229E.

HCoV-229E, an α-CoV, is an important cause of respiratory viral infections in high-risk infants (Sizun et al., 2001). Saikosaponins are a group of oleanane derivatives that have been isolated from some medicinal plants such as *Heteromorpha* spp. (Recio et al., 1995). Saikosaponins (A (35), D (36), B2 (37) and C (38)) were found to possess antiviral activity on HCoV-229E at concentrations of 0.25–25 µM. Saikosaponin B2 (**37**) possessed strongest activity (IC_50_ = 1.7 ± 0.1 µM). Saikosaponin B2 (**37**) inhibited HCoV-229E viral infection by inhibiting attachment of viruses to cells, blocking viral penetration into cells, and also interfering with the early stages of viral replication (Cheng et al., 2006). Also, the efficacy of aqueous and hydromethanolic extracts from stem bark and leaves of three mulberry species (*Morus alba* L. var*. alba, Morus alba* L. var. *rosa* and *Morus rubra* L.) against HCoV-229E has been investigated. Leaf water-alcohol extracts had the best antiviral activity against human coronavirus 229E (Thabti et al., 2020). The structures of natural products against HCoV-229E are shown in Figure 2.

#### Natural Products Against SARS-CoV-2

As mentioned before, SARS-CoV-2 can cause severe respiratory illness and even death. An in silico investigation revealed that some Indian herbal plant extracts inhibited the SARS-CoV-2 main protease. Highly promising inhibition was shown by extracts of harsingar, *Aloe vera* (L.) Burm. f., and giloy (*Tinospora cordifolia* (Willd.) Hook. f. & Thomson) (Srivastava et al., 2020).

Currently, there is no vaccine or effective antiviral treatment available for COVID-19. In this regard traditional medicine and natural compounds can be taken into consideration as one of the treatment modalities. Lianhuaqingwen (LH) is a traditional Chinese medicine. The antiviral effect of the LH Capsule (LHC) against influenza has been reported. It can reduce the duration of the illness and the duration of viral shedding (Duan et al., 2011). Recently the antiviral and anti-inflammatory activity of LHC against SARS-CoV-2 was investigated *in vitro*. Results showed that LHC significantly inhibited the replication of SARS-COV-2, affected virus morphology, and exhibited anti-inflammatory activity *in vitro* (Runfeng et al., 2020). These multiple beneficial effects are promising and indicate that further intensive research on this preparation is needed.

Terpenoids are a structurally diverse group of plant secondary metabolites (Cheng et al., 2007) and several such compounds have been proven to be effective against SARS-CoV-2. Some terpenoids, namely ursolic acid (**39**), oleanolic acid (**40**) and carvacrol (**41**) were shown to be potential inhibitors of the main protease of SARS-CoV-2 using integrated molecular modeling approaches (Kumar et al., 2020). According to another computational investigation, three natural compounds, digitoxigenin (**42**), β-eudesmol (**43**), and crocin (**44**), had good activity against the main protease of coronavirus and can be proposed as inhibitors of the COVID-19 main protease (Aanouz et al., 2020). Artemisin (**45**) and a limonoid, 6-α-acetoxygedunin (**46**), as well as glycyrrhizin (**47**) are terpenoids which showed potential inhibition against the COVID-19 main protease active site and ACE2 in an *in silico* study (Chen and Du, 2020; Khandelwal and Sharma, 2020; Omaret al., 2020) Also, among the several natural products screened by docking analysis, glycyrrhizin (**47**) and some other natural products including tryptanthrine, rhein and berberine were found to exhibit a higher degree of interaction with the COVID-19 main protease (Narkhede et al., 2020).

Recently over 180,000 natural product-based compounds from different species of animals and plants were screened against the COVID-19 main protease and the ADME properties evaluated (unpublished data). According to the results, twenty compounds were selected and introduced as new potential inhibitors. Compound (48), comprising an alkaloid structure, showed a strong binding affinity to the crucial residues of COVID-19 main protease. Additionally, the excellent ADME properties strengthened the potential of this compound to be a promising drug for the treatment of COVID-19, but this is still under evaluation. Monajjemi and co-workers investigated interactions between four natural compounds, extracted from plants of the province Gillan in Iran, and three receptors including human ACE2, COVID-19 main protease, and SARS-CoV nsp12 polymerase. Results showed that cytarabine (**49**), a compound obtained from Chuchaq (*Eryngium planum* L.), and matrine (**50**) from Trshvash (*Oxalis corniculata* L.), bind to those receptors with lower energies compared to the respective reference compounds (Monajjemi et al., 2020). Khandelwal and Sharma (2020) reported that an alkaloid named echitamine (**51**) had a strong inhibitory effect on ACE2. Another alkaloid named nicotianamine (**52**), seemed to have the potential to block the entry of 2019-nCoV into host cells by binding to ACE2 (Chen and Du, 2020). Hydroxy-chloroquine and chloroquine, synthetic derivatives of quinine, showed positive activities against SARS-CoV-2 *in vitro* and *in vivo*. Quinine is an alkaloid extract from the bark of *Remija* and *Cinchona* (Rubiaceae) species (Gautret et al., 2020). Moreover, some flavonoids including baicalin (**53**), scutellarin (**54**) and hesperetin (**55**) exhibited promising anti-2019-nCoV effects. They have the potential to bind to ACE2 and block the entry of 2019-nCoV into host cells (Chen and Du, 2020).

A computational *in vitro* and *in vivo* study on citrus peels showed that naringin (**56**) could have potential activity in preventing cytokine storms of COVID-19 and naringin (**56**) and hesperetin (**55**) had strong binding affinity to the ACE2 (Cheng et al., 2020a; Cheng et al., 2020b). Additionally, among all the flavonoids available in the traditional Chinese herb, *Exocarpium Citri grandis,* naringin (**56**) possessed the greatest potential for application in reducing the respiratory symptoms caused by COVID-19. In this study some notable features of naringin (**56**) were revealed, namely that it could improve lung function, alleviate acute lung injury, have effects in attenuating pulmonary fibrosis, and enhance the antiviral immune response through its catabolite HPPA (Su et al., 2020).

An *in silico* study proved that quercetin (**6**), hispidulin (**3**), and cirsimaritin (**57**) exhibited better potential inhibition than hydroxy-chloroquine potential inhibition against the COVID-19 main protease active site and ACE2 (Omar et al., 2020). Moreover, according to the results of another *in silico* study in which 8,000 small molecule candidates of known drugs and natural products were screened, quercetin (**6**) was among the top five most potent compounds for binding strongly to the S-protein ACE2 (Smith and Smith, 2020). Based on the results of a computational study in which a library of phenolic natural compounds (comprising 80 flavonoids) was investigated against SARS-CoV-2 main protease, some other flavonoids including hesperidin (**58**), rutin (**8**), diosmin (**59**) and apiin (**60**) were found to have the potential to bind to the active site. Hesperidin was reported to have the best binding affinity to the main protease (Adem et al., 2020). Also, in another *in silico* study, rutin (**8**) showed notable inhibitory activity against SARS-CoV-2 main protease (Das et al., 2020).

Despite the notable effects of quercetin (**6)** and its derivatives, there are some issues which should be considered. Quercetin and its glucosides found in plants were preferred for interaction with proteins (Day et al., 2000). It has been proven that, despite administration of a high oral dose of quercetin, plasma contained traces of the glycoside due to biotransformation in the gastrointestinal tract (Mullen et al., 2004). Thus, the required concentration for quercetin to perform as an inhibitor of the SARS-CoV-2, Viral Spike Protein/ACE is not reachable by oral administration. However, by using a nasal spray containing quercetin glucosides in a suitable form, the appropriate concentration can be delivered to the active sites (Zhao et al., 2019). Later in 2020, administration of quercetin was recommended to be done directly through alternative routes such as a nasal or throat spray to be effective in clinical trials (Williamson and Kerimi, 2020).

As mentioned earlier, there is more than one host-cell receptor which has been reported to be recognized by the viral spike protein. HSPA5, also termed GRP78 or BiP, is one of these receptors in the cell-surface. Some active components found in some natural products have been tested against HSPA5 *in silico.* Based on the obtained results, some phytoestrogens (diadiazin, genistein, formontein, and biochanin A) and estrogens have the best binding affinities to HSPA5 (Elfiky, 2020). According to the results of a computational study there are several natural molecules like δ-viniferin (**66**), myricitrin (**61**), taiwanhomoflavone A, lactucopicrin 15-oxalate, nympholide A, afzelin, biorobin, hesperidin, and phyllaemblicin B that strongly bind to the SARS-CoV-2 M^Pro^. The flavonoid myricitrin (**61**) showed strong binding with SARS-CoV-2 M^Pro^ and also high solubility and bioavailability. Interestingly, this compound also showed strong binding with other potential targets of SARS-CoV-2 infection like viral receptor ACE-2 and RNA dependent RNA polymerase (RdRp) (Joshi et al., 2020). An *in silico* study with a focus on three target proteins important in the life cycle of SARS-CoV-2, namely Spike glycoprotein, main protease and RNA-dependent RNA-polymerase was performed. The results showed that silybin (**62**), an active constituent found in *Silybum marianum*, showed binding affinity with targets in SARS-CoV-2. Also, withaferin A from *Withania somnifera* showed significant binding to the target proteins (Pandit and Latha, 2020). According to many studies, PLpro and 3CLpro can be considered as important targets for antiviral drugs against coronaviruses. About 38 drugs and analogues with antiviral activity and 55 of natural origin were screened for inhibitory activities against PLpro and 3CLpro. The results showed that saikosaponin D (**36**) possessed the highest affinity to 3 CL-PRO; Conversely, amentoflavone (**63**) seemed to be a promising inhibitor of PLpro (Contreras-Puentes and Alvíz-Amador, 2020). Molecular docking studies of 32,297 potential anti-viral phytochemicals/traditional Chinese medicinal compounds against 3CL^pro^ of SARS-CoV-2 were performed. The results showed that two flavonoids including 5,7,3′,4′-tetrahydroxy-2’-(3,3-dimethylallyl) isoflavone (**64**) and myricitrin (**61**) and a compound named methyl rosmarinate (27) had inhibitory effects against 3CL^pro^ of SARS-CoV-2 (Tahir ul Qamar et al., 2020).

A combination of the HPLC-Q-Exactive-MS/MS method with molecular docking showed that active alkaloids of the dried roots and rhizomes of *Veratrum nigrum* L (Agsirga) could block the binding of 2019-nCoV S-protein and ACE2. Agsirga is a traditional Mongolian medicine commonly used to treat tumor and cancer (Cheng et al., 2020a; Cheng et al., 2020b).

Curcumin (**65**), the principal curcuminoid in the rhizome of *Curcuma longa* L., has been reported to be active against the COVID-19 main protease and ACE2 (Omar et al., 2020). This compound has been proven to bind to the active site of SARS-CoV-2 main protease (Das et al., 2020). Also, δ-viniferin (**66**) showed strong binding with the SARS-CoV-2 main protease and strong binding affinity to ACE-2 and RNA dependent RNA polymerase (RdRp) (Joshi et al., 2020).

As mentioned before¸ TMPRSS2 is essential for viral spread and pathogenicity and a TMPRSS2 inhibitor might constitute a treatment option. A virtual screening of natural products against TMPRSS2 indicated that among compounds with promising features, geniposide (**67**) can be considered as the best drug candidate for drug development. Geniposide (**67**) is an iridoid found in the *Gardenia* genus (Rubiaceae) and is endemic in Central America and China (Rahman et al., 2020).

Eight compounds found in rhizomes of *Alpinia officinarum* and ginger were identified as potential inhibitors of SARS-CoV-2 PLpro. Binding affinities to closed and open conformer of PLpro were evaluated. Based on the obtained results, five compounds from the rhizome of *Alpinia officinarum* were identified. Compound **68** binds with the highest affinity to the open conformer of SARS-Cov-2 PLpro and three compounds including 8-gingerol, 10-gingerol and 6-gingerol from ginger were identified to be potent inhibitors of PLpro (Goswami et al., 2020).

**FIGURE 3:**
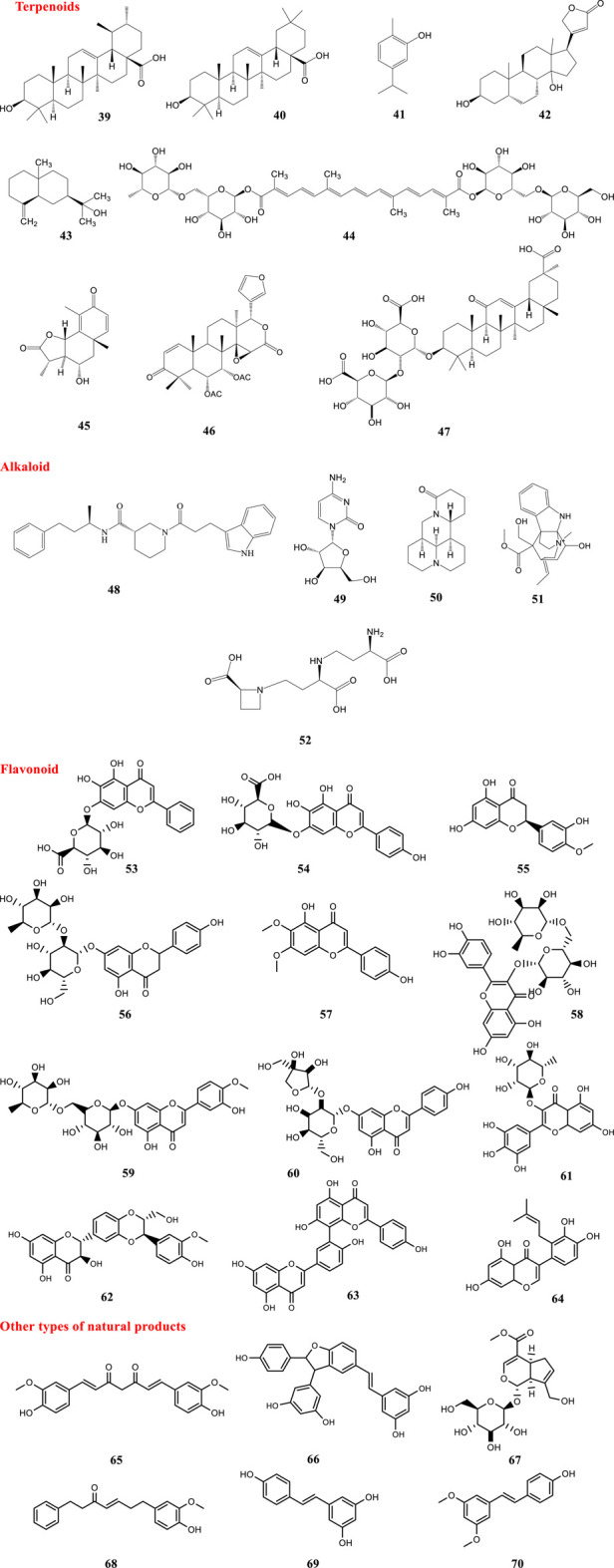
Structures of natural compounds against SARS-CoV-2.

Resveratrol (**69**) is a stilbenoid commonly found in *Vitis* species (grapes), red wine and some other plants (Akinwumi et al., 2018). There have been some studies assessing the impact of resveratrol on SARS-CoV-2 ACE2 activity. An *in silico* study revealed that resveratrol showed significant binding with ACE2 over other tested stilbenoids in the study (Wahedi et al., 2020). Also, resveratrol (**69**) and pterostilbene (**70**) inhibited SARS-CoV-2 infection in a Vero-E6 model. The compounds interfered with the viral infectious cycle and significantly inhibited COVID-19 infection in primary human bronchial epithelial cells cultured under air liquid interface conditions (ter Ellen et al., 2020). The structures of natural products against SARS-CoV-2 are demonstrated in Figure 3.

The efficacy of some natural compounds and medicinal plants has been tested clinically. Based on biological therapeutic activities, resveratrol (**69**) has been suggested as a potential treatment adjunct for COVID-19 (Filardo et al., 2020; Marinella, 2020). Resveratrol (**69**) can reduce copper (II) to copper (I) thereby generating highly unstable free-radicals which can degrade cell-free chromatin and can lead to prevention of endotoxin sepsis in mice. In one study, a nearly two-fold reduction in mortality following treatment with resveratrol-copper was observed. In this study thirty patients with severe COVID-19 received, in addition to standard care, resveratrol (**69**) and copper at doses of 5.6 mg and 560 ng, respectively orally, once every 6 h, until discharge or death (Mittra et al., 2020). Lianhua Qingwen prescription (capsules or granules) is an innovative patented Chinese medicine that is composed of 11 herbs with gypsum and menthol, and this preparation had antiviral activities against viral respiratory infections. It includes *Forsythia suspensa* (Thunb.) Vahl (Lianqiao), *Lonicera japonica* Thunb (Jinyinhua), *Ephedra sinica* Stapf (Mahuang), Armeniacae Amarum Semen (Kuxingren), Gypsum Fibrosuum (Shigao), *Isatis tinctoria* L (Banlangen), Dryopteridis Crassirhizomatis Rhizoma (Mianmaguanzhong), *Houttuynia cordata* Thunb (Yuxingcao), *Pogostemon cablin* (Blanco) Benth (Guanghuoxiang), *Rheum palmatum* L (Dahuang), *Rhodiola rosea* Linn (Hongjingtian), *Mentha haplocalyx* Briq (Bohe), *Glycyrrhiza uralensis* Fisch (Gancao) with a herbal ratio of 170 g: 170 g: 57 g: 57 g: 170 g: 170 g: 170 g: 170 g: 57 g: 34 g: 57 g: 5 g: 57 g, which is recorded in the Chinese Pharmacopeia. A study of 284 patients who received Lianhua Qinwen Capsules in combination with basic treatment showed the recovery rate in treatment group was significantly higher than the control group. Time of recovery of symptoms, fever, fatigue, and coughing was remarkably shorter in treatment (Hu et al., 2020). Also, another study reported that Lianhua Qingwen Granules could inhibit fever and cough, reduce their duration and improve individual symptoms (Yao et al., 2020). Also, some other traditional Chinese medicines were found to improve patient recovery (Luo et al., 2020). Results showed that a quadruple combination Ribavirin, Lopinavir/ritonavir, Umifenovir, and Lianhua Qingwen capsule could result in an improvement in abnormal coagulation and leukocytes, with a better prognosis (Li et al., 2020).

#### Natural Products Against SARS-CoV

SARS is a respiratory illness caused by severe acute respiratory syndrome coronavirus (SARS-CoV) (Drosten et al., 2003). There is evidence that supports medicinal plants and natural products in having beneficial effects in the treatment or prevention of SARS. In 2005, a study revealed that taking a modified formula of two Chinese herbal medicines (Yupingfeng Powder and Sangju Decoction) resulted in the prevention of SARS-CoV (Lau et al., 2005). None of the health care workers using the supplement contracted SARS, in comparison to 0.4% of health care workers who did not use the supplement. Improvements in influenza-like symptoms in addition to quality of life measurements were also noted among herbal supplement users (Lau et al., 2005).

As mentioned before, PLpro can be considered as an important target for antiviral agents (Lenschow et al., 2007). *Paulownia tomentosa* (Thunb.) Steud., a traditional Chinese medicine, is a polyphenol-rich plant (Šmejkal et al., 2007). Flavonoids possess hydrophobic aromatic rings and hydrophilic hydroxyl groups, so they have a wide range of binding affinity to SARS-CoV 3CLpro. The binding affinity and mode of the chromen-4-one moiety depends on the presence of carbohydrate groups (Jo et al., 2020). Many small molecules capable of targeting PLpro have been isolated from the methanol extracts of the fruits of the *Paulownia* tree (*P. tomentosa*). Five new geranylated flavonoids, including tomentin A-E (**71–75**) were among these compounds. They all contain a 3,4-dihydro-2H-pyran group. Most isolated compounds (**71**–**82**) were evaluated as PLpro inhibitors with IC_50_ values ranging between 5.0 and 14.4 μM. All new compounds with a dihydro-2H-pyran group in their structure showed better inhibition than their parent compounds (Cho et al., 2013). According to the results of an induced-fit docking experiment, the presence of an additional 8-hydroxyl group of herbacetin (**83**) was anticipated to be critical for its high binding affinity around the S1 and S2 sites. The occupation of the S1 and S2 sites by carbohydrate groups of rhoifolin (**84**) and pectolinarin (**85**) was expected to be an additional way to achieve a high affinity to SARS-CoV 3CLpro in glycosylated flavonoids (Jo et al., 2020). In 2005, a natural product library comprising 720 compounds was screened for inhibitory activity against 3CLPro. Also the 3CLPro-inhibitory activity of extracts from different types of teas, including green tea, oolong tea, Puer tea and black tea was further investigated. Two types of tea, including Puer and black tea, showed inhibitory activities against 3CLPro. Theaflavin-3,3′-digallate (TF3) (**86**), a known ingredient in teas, was found to be a 3CLPro inhibitor (Chen et al., 2005). Moreover, some biflavonoids from *Torreya nucifera* (L.) Siebold & Zucc. are reported to be active against the 3CLpro. The biflavone amentoflavone (**87**) was the most potent inhibitor of the 3CLpro (Ryu et al., 2010). Also, quercetin (**6**), epigallocatechin gallate (**10**), and gallocatechin gallate (GCG) (**88**) isolated from the yeast *Pichia pastoris* displayed good inhibition of 3CLpro (Nguyen et al., 2012). Quercetin-3-β-galactoside (**89**) (IC_50_ = 42.79 ± 4.97 μM) was identified as a potent inhibitor of SARS-CoV 3CLpro by molecular docking studies and enzymatic inhibition assays. Additionally, the structure–activity relationship of eight new derivatives of quercetin (**6**) was investigated with the help of molecular modeling. Results revealed that the bioactivity of the derivatives was reduced by the removal of the 7-hydroxy group of the quercetin (**6**) moiety and acetoxylation of the sugar moiety (Chen et al., 2006). Xanthoangelol E (**88**), isolated from *Angelica keiskei* (Miq.) Koidz., is a chalcone with a perhydroxyl group in its structure. Compound **90** showed 3CLpro and PLpro inhibitory activity with IC_50_ values of 11.4 and 1.2 μM. Further protein-inhibitor mechanistic analysis revealed that inhibition properties of chalcones to the SARS-CoV 3CLpro seem to be competitive, whereas non-competitive inhibition was observed with the SARS-CoV PLpro (Park et al., 2016). Papyriflavonol A (**91)** is a polyphenol isolated from *Broussonetia papyrifera* (L.) L’Hér. ex Vent. It acted as an inhibitor of PLpro with an IC_50_ value of 3.7 μM (Park et al., 2017). Additionally, six polyphenols, bavachinin (**90**), neobavaisoflavone (**93**), isobavachalcone (**94**), 4′-O-methylbavachalcone (**95**), and corylifol A (**96**), were isolated from *Psoralea corylifolia* L (current scientific name Cullen corylifolium (L.) Medik.). These phytochemicals were identified as replication inhibitors of SARS-CoV by inhibiting PLpro in a dose-dependent manner with IC_50_ ranging between 4.2 and 38.4 µM (Kim et al., 2014). Myricetin (**97**) and scutellarein (**98**) are two naturally-occurring flavonoids. Both compounds act as potent inhibitors of the SARS-CoV helicase protein *in vitro* by affecting the ATPase activity (Yu et al., 2012).

Some natural products showed moderate inhibitory activity against SARS-CoV, for instance procyanidin B1 (**99**), procyanidin A2 (**100**), and cinnamtannin B1 (**101**) extracted from Cinnamon cortex (dried bark of *Cinnamomum verum* J. Presl) and the ethanol extract of Cinnamon cortex have been reported with a low or moderate anti-SARS-CoV activity (Zhuang et al., 2009).

Moreover, some terpenoids named quinone-methide triterpenes including celastrol (**102**), pristimerin (**103**), tingenone (**104**), and iguesterin (**105**) isolated from *Tripterygium regelii* Sprague & Takeda exerted inhibitory activity against SARS-CoV 3CLpro (Ryu et al., 2010). The abietane type diterpenoids isolated from the ethanol extract of *Salvia miltiorrhiza* Bunge such as tanshinone IIA (**106**), tanshinone IIB (**107**), methyl tanshinonate (**108**), cryptotanshinone (**109**), tanshinone I (**110**), dihydrotanshinone I (**111**), and rosmariquinone (**112**)) were identified as inhibitors of the SARS-CoV 3CLpro and PLpro. The inhibitory activity of all seven compounds (**106**–**112**) was considerable (ranging from 0.8 to 30.0 μM) and an improvement in the inhibition was observed with preincubation with the PLpro. Interestingly, the inhibition was selective because no inhibitory effects against other proteases were observed (Park et al., 2012).

According to the results of an investigation on 224 phytocompounds in 2017, 20 compounds including ten diterpenoids, two sesquiterpenoids, two triterpenoids, five lignoids and one curcumin were identified to be active against SARS-CoV in a cell-based assay of cytopathogenic effect on Vero E6 cells. All compounds exhibited significant inhibition on SARS-CoV 3CLpro (Wen et al., 2007). Glycyrrhizin (**47**) is the principal triterpenoid isolated from licorice (*Glycyrrhiza glabra* L.) roots. In 2003, the results of an investigation showed that glycyrrhizin (**47**) acted as a potent inhibitor of SARS-CoV replication in Vero cells with a selectivity index of 67. Although glycyrrhizin (**47**) had a low selectivity index, it was a significantly potent inhibitor of replication of all the viruses tested and few toxic effects of glycyrrhizin (**47**) were reported (Cinatl et al., 2003). In 2004, some commercial antiviral agents and purified compounds extracted from traditional Chinese medicinal herbs were screened against SARS-CoV. Glycyrrhizin (**47**) and some other compounds including interferon-beta-1a, leukocytic interferon-alpha, ribavirin, rimantadine, lopinavir and baicalin showed antiviral activity against SARS-CoV. The two interferons were only effective when the cells were pre-incubated with the drugs 16 h before viral inoculation, and antiviral activity depended on the cell lines used. Vero, Vero E6, and fRhK-4 cell lines were used in this investigation. Inhibitory activities were not observed for artesunate, glycyrrhizin (**47**) and chlorogenic acid in fRhK-4 cell line. Ribavirin, baicalin and lopinavir were less active in the Vero-E6 cell line while glycyrrhizin, rimandatine, leukocytic interferon-alpha and interferon-beta were more active. Since antiviral activity could be shown for most of the agents in Vero cells, Vero cells were used instead of Vero E6 or fRhK-4 cells for the plaque reduction assay (Chen et al., 2004). Aescin (**113**), the major active principle from the *Aesculus hippocastanum* L (horse chestnut), was reported to have inhibitory activity against SARS-CoV with EC50 value of 6 μM and CC_50_ value of 15 μM (SI = 2.5) in a cell based assay (Wu et al., 2004).

Some alkaloids have been reported to be active against SARS-CoV. Six cinnamic amides (**114**–**119**) were isolated from *Tribulus terrestris* L. fruits. These compounds were proven to be active against SARS-CoV PLpro with IC_50_ values in the range 15.8–70.1 µM (Song et al., 2014). Indigo (**120**) is an alkaloid isolated from *Isatis indigotica* with the ability to block the cleavage processing of the 3CLpro (Lin et al., 2005). In 2005, antiviral activities of more than 200 Chinese medicinal herb extracts against SARS-CoV were evaluated. Among all extracts, the ethanol extract of *Lycoris radiata* (L’Hér.) Herb. had the most potent antiviral activity against SARS-CoV. The process of further purification in order to identify the active compound led to the isolation and identification of an alkaloid, lycorine (**121**), as a potent antiviral compound against SARS-CoV with EC50 ranging from 2.4 ± 0.2 to 88.2 ± 7.7 μg/ml. In the cytotoxicity assay, this compound had a CC50 value of 14,980.0 ± 912.0 nM, and a selective index (SI) greater than 900 (Li et al., 2005a; Li et al., 2005b; Li et al., 2005c).

Aloeemodin (**122**) and hesperetin (**55**) are two phenolic compounds, isolated from *Isatis indigotica* Fortune ex Lindl. root extract, that inhibit cleavage activity of the 3CLpro in dose-dependent manners. Sinigrin (**123**), and beta-sitosterol (**124**) are other isolated compounds with the ability to block the cleavage processing of the 3CLpro in cell-free and cell-based assays (Lin et al., 2005). The inhibitory action of dieckol (**125**), a phlorotannin isolated from the algal species *Ecklonia cava*, against SARS-CoV 3CLpro was investigated. Dieckol (**125**) (IC_50_ = 2.7 μM) showed remarkable inhibitory activity against (**125**) SARS-CoV 3CLpro cell-free cleavage. Additionally, in silico molecular docking simulation of dieckol (**125**) was performed to evaluate its interactions with protein residues in the original ligand-binding site. The findings from docking experiments confirmed the important inhibitory effect of this compound against SARS-CoV 3CLpro (Park et al., 2013). Psoralidin (**126**) is a natural phenolic compound isolated from *Psoralea corylifolia* L. which has been proved to inhibit PLproof SARS-CoV (Kim et al., 2014). Emodin (**127**) is an anthraquinone which significantly blocked the S protein, and also ACE2 interaction, in a dose-dependent manner. It was derived from the genera *Rheum officinale* Baill. and *Polygonum multiflorum* Thunb (current scientific name Reynoutria multiflora (Thunb.) Moldenke). which were identified to be active against SARS-CoV with IC_50_ values ranging from 1 to 10 μg/ml. Emodin (**127**) inhibited the infectivity of S protein-pseudotyped retrovirus to Vero E6 cells (Ho et al., 2007).

**FIGURE 4:**
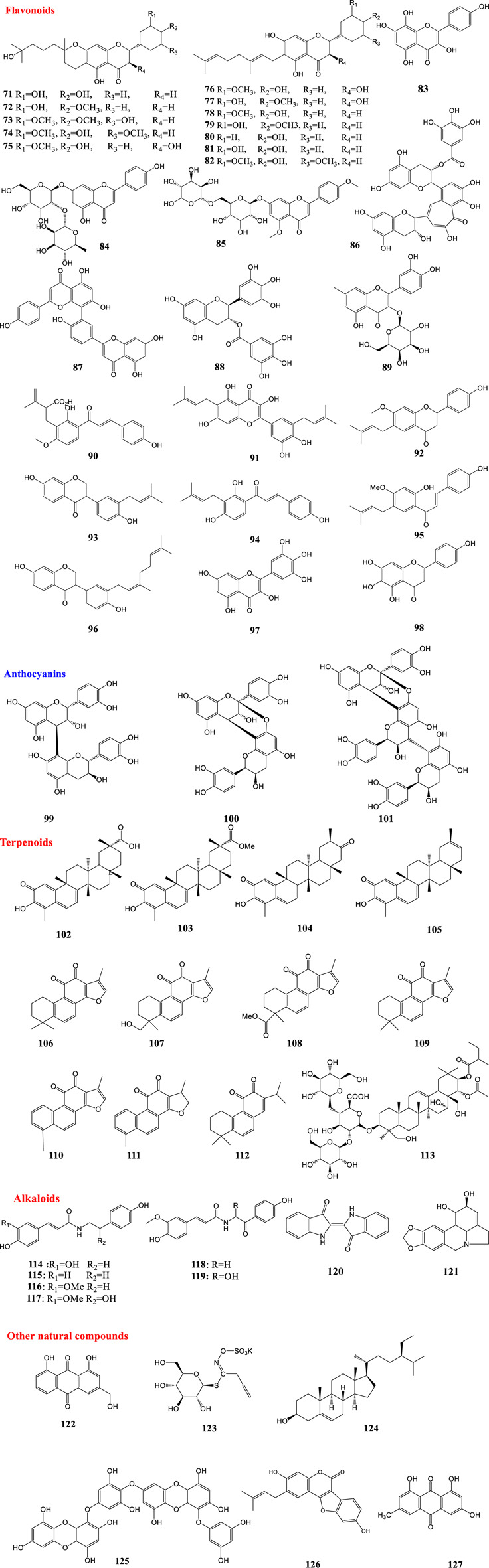
Structures of natural products against SARS-CoV.

Wu and co-workers highlighted potential inhibitors against SARS-CoV and identified numerous potent SARS-CoV inhibitors through screening of a library of natural products. These compounds showed inhibitory activity against viral replication. Some active compounds were able to inhibit the 3CL protease and viral entry (Wu et al., 2004). Moreover, the potential activity of the extracts of some plants against SARS-CoV has been studied. *Houttuynia cordata* Thunb. water extract exhibited significant inhibitory effects on SARS-CoV 3CLpro (Lau et al., 2008). There is evidence of *H. cordata* protecting cells against other coronaviruses as well (Yin et al., 2011). Methanol extracts of Cimicifuga rhizoma, Phellodendron cortex, and Sophora subprostrata radix have been identified as inhibitors of RNA synthesis and N and S expression *in vitro* (Kim et al., 2008). The structures of mentioned natural compounds against SARS-CoV are shown in Figure 4.

#### Natural Products Against Middle East Respiratory Syndrome-Coronavirus

MERS-CoV, belonging to β-CoVs, is mainly endemic in the Middle East but it can also spread outside this region. MERS-CoV causes severe human respiratory disease with a case fatality rate close to 40% (Arabi et al., 2017). In 2018, 502 compounds derived from natural products, either animal or plant, were screened for their ability to block MERS-CoV entry. Of all tested compounds, dihydrotanshinone (**128**) exhibited antiviral activity against MERS-CoV in the post-attachment assay. Also, it showed antiviral activities in the pre-attachment assay. Therefore dihydrotanshinone (**128**) may have dual inhibitory effects that block virus entry and inhibit S proteins (Kim et al., 2018).

The binding affinity of four compounds isolated from *Coriandrum sativum* L. with six proteins of MERS-CoV was evaluated via in silico methods. Virtual screening and molecular docking results showed that dodecanal (**129**) had the highest binding affinity with all the selected proteins (Rao et al., 2018).

**FIGURE 5:**
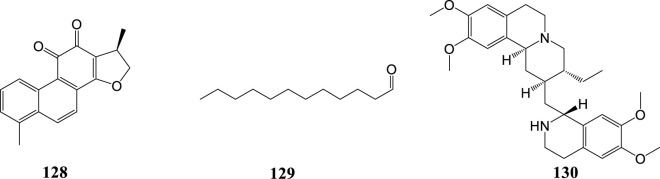
Structures of natural products against MERS-CoV.

In 2019, a high-throughput screening of potential inhibitors of CoVs *in vitro* resulted in the identification of seven compounds as inhibitors of the replication of CoVs. Among the seven inhibitors, the alkaloid emetine (**130**) exhibited the strongest anti-MERS-CoV activity with an EC50 value of 0.34 μM and SI of 9.06. It acted as an entry inhibitor blocking MERS-CoV-S-mediated infection (Shen et al., 2019). Natural products against coronaviruses are summarized in Table 2 and their structures are shown in Figure 5.

**TABLE 2:** Natural products against coronaviruses.

Virus type	Plant/solvent	Compounds	Effect	Ref
HCOV-229E	*Heteromorpha* spp.	Saikosaponins A, D, B_2_, C **(35–38)**	Inhibiting viral attachment, blocking viral penetration, interfering with viral replication	Cheng et al. (2006)
HCOV-229E	*Morus alba* L. var. *alba, Morus alba* L. var. *rosa, Morus rubra* L., water/methanol	Crude extract	Reducing the viral titer and cytopathogenic effects	Thabti et al. (2020)
SARS-CoV-2	*—*	Ursolic acid (**39)**, Oleanolic acid (**40**), Carvacrol (**41**)	Inhibiting the main protease	Kumar et al. (2020)
SARS-CoV-2	*In silico*	Digitoxigenin **(42)**, β-cudesmol **(43)**, Crocin **(44)**	Inhibiting SARS-CoV-2 main protease	Aanouz et al., (2020)
SARS-CoV-2	*In silico*	Artemisin (**45**)	Inhibition against COVID-19 main protease active site and ACE2	Omar et al. (2020)
SARS-CoV-2	*In silico*	6-α-acetoxygedunin (**46**)	Inhibiting ACE2	Khandelwal and Sharma (2020)
SARS-CoV-2	—	Glycyrrhizin **(47)**	Bind to ACE2 and block the entry	Chen and Du (2020)
SARS-CoV-2	*(In silico)*	An alkaloid **(48)**	Inhibiting SARS-CoV-2 main protease	Under evaluation
SARS-CoV-2/SARS- CoV	*Eryngium planum* L	Cytarabine **(49)**	Binding to SARS-CoV-2 main protease and SARS-CoV nsp12 polymerase	Monajjemi et al. (2020)
SARS-CoV-2/SARS- CoV	*Oxalis corniculata* L	Matrine **(50)**	—	Monajjemi et al. (2020)
SARS-CoV-2	*In silico*	Echitamine (**51**)	Inhibiting ACE2	Khandelwal and Sharma (2020)
SARS-CoV-2	—	Nicotianamine **(52)**, Baicalin **(53),** Scutellarin **(54)**, Hesperetin **(55)**	Bind to ACE2 and block the entry	Chen and Du (2020)
SARS-CoV-2	*Citrus wilsonii Tanaka*	Hesperetin **(55),** Naringin (**56**)	Inhibiting ACE2	Cheng et al. (2020a); Cheng et al. (2020b)
SARS-CoV-2	*In silico, Exocarpium Citri grandis*	Naringin (**56**)	Binding affinity to ACE2 and main protease	Su et al. (2020)
SARS-CoV-2	*In silico*	Phytoestrogens and estrogens	Binding affinity to HSPA5	Elfiky (2020)
SARS-CoV-2	*In silico*	Quercetin (**6**), Hispidulin (**3**), Cirsimaritin (**57**)	Inhibition against COVID-19 main protease active site and ACE2	Omar et al. (2020)
SARS-CoV-2	*In silico*	Hesperidin (**58**), Rutin (**8**), Diosmin (**59**), Apiin **(60**)	Inhibiting main protease	Adem et al. (2020)
SARS-CoV-2	*In silico*	Myricitrin **(61)**	Strong binding affinity to ACE-2 and RNA dependent RNA polymerase	Joshi et al. (2020)
SARS-CoV-2	*In silico Silybum marianum*	Silybin **(62)**	Inhibitory effect on spike glycoprotein, main protease and RNA-dependent RNA-polymerase	Pandit and Latha (2020)
SARS-CoV-2	*In silico*	Amentoflavone **(63)**	Inhibiting PL^pro^	Contreras-Puentes and Alvíz-Amador (2020)
SARS-CoV-2	*In silico*	5,7,3′,4′-tetrahydroxy-2’-(3,3-dimethylallyl) isoflavone **(64**), Myricitrin **(61),** Methyl rosmarinate **(27)**	Inhibiting 3CL^pro^	Tahir ul Qamar et al. (2020)
SARS-CoV-2	*In silico*	Curcumin **(65)**	Inhibition against COVID-19 main protease active site and ACE2	Omar et al. (2020)
SARS-CoV-2	*In silico*	δ-Viniferin **(66)**	Strong binding affinity to ACE-2 and RNA dependent RNA polymerase	Joshi et al. (2020)
SARS-CoV-2	*In silico*	Geniposide **(67)**	Inhibitory effect on TMPRSS2	Rahman et al. (2020)
SARS-CoV-2	*In silico Alpinia officinarum* and ginger	8-Gingerol, 10- Gingerol and 6-Gingerol and Compound **(68)**	Inhibitors of PLpro	Goswami et al. (2020)
SARS-CoV-2	*In silico*	Saikosaponin D **(36)**	Inhibiting 3CL^Pro^	Contreras-Puentes and Alvíz-Amador (2020)
SARS-CoV-2	*In cilico*	Resveratrol **(69)**	Inhibiting ACE2	Wahedi et al. (2020)
SARS-CoV-2	*Vitis* species (grapes), red wine	Resveratrol (**69**) and pterostilbene (**70**)	Interfere with the COVID-19 infection in Vero-E6 model	ter Ellen et al., (2020)
SARS-CoV	*Paulownia tomentosa* (Thunb.) Steud./methanol	Tomentin A-E **(71–75)**	Inhibiting PL^pro^	Cho et al. (2013)
SARS-CoV	*Tribulus terrestris* L.*/*ethyl acetate, methanol, water	Cinnamic amides **(76–82)**	Inhibiting PL^pro^	Song et al. (2014)
SARS-CoV	*(In silico)*	Herbacetin **(83)**, Rhoifolin **(84)**, Pectolinarin **(85)**	Binding to S1 S and S2 sites of 3CLpro	Jo et al. (2020)
SARS-CoV	*(In silico)*	Theaflavin-3,3′-digallate (TF3) **(86)**	Inhibiting 3CL^Pro^	Chen et al., (2005)
SARS-CoV	Torreya nucifera (L.) Siebold & Zucc	Amentoflavone **(87)**	Inhibiting 3CL^Pro^	Ryu et al. (2010)
SARS-CoV	*Pichia brastoris*	Quercetin **(6)**, Epigallocatechin gallate **(10)**, Gallocatechin gallate **(88)**	Inhibiting 3CL^pro^	Nguyen et al. (2012)
SARS-CoV	*(In silico)*	Quercetin-3-β-galactoside **(89)**	Inhibitor of SARS-CoV 3CL^pro^	Chen et al., (2006)
SARS-CoV	*Angelica keiskei* (miq.) koidz*.*/ethanol	Xanthoangelol E **(90)**	Inhibitor of SARS-CoV 3CL^pro^ and PL^pro^	Park et al. (2016)
SARS-CoV	*Broussonetia papyrifera* (L.) L’Hér. ex Vent /ethanol	Papyriflavonol A **(91)**	Inhibitor of PL^pro^	Park et al. (2017)
SARS-CoV	*Psoralea corylifolia* L. (Cullen corylifolium (L.) Medik.)/ethanol	Bavachinin **(92)**, Neobavaisoflavone **(93)**, Isobavachalcone **(94**), 4′-O-Methylbavachalcone **(95)**, Corylifol A **(96)**	Inhibiting PL^pro^	Kim et al. (2014)
**SARS-CoV**	—	Myricetin **(97)**, scutellarein **(98)**	Inhibiting the SARS-CoV helicase protein by affecting the atpase	Yu et al. (2012)
SARS-CoV	Cinnamon cortex, cinnamon cortex*/*methanol	Procyanidin B1 (**99**), procyanidin A2 (**100**), cinnamtannin B1 **(101)**	Inhibitory activity against SARS-CoV	Zhuang et al., (2009)
SARS-CoV	Tripterygium regelii Sprague & Takeda/ethanol	Celastrol **(102)**, Pristimerin **(103)**, tingenone **(104)**, Iguesterin **(105)**	Inhibitor of SARS-CoV 3CL^pro^	Ryu et al. (2010)
SARS-CoV	Salvia miltiorrhiza Bunge/ethanol	Tanshinone IIA **(106)**, Tanshinone IIB **(107)**, Methyl tanshinonate **(108)**, Cryptotanshinone **(109)**, Tanshinone I **(110)**, Dihydrotanshinone I **(111)**, Rosmariquinone **(112)**	Inhibition in preincubation with the PL^pro^	Park et al. (2012)
SARS-CoV	*Aesculus hippocastanum* **L**	Aescin **(113)**	Inhibitory activity in a cell based assay	Wu et al. (2004)
SARS-Cov	*Tribulus terrestris* L. fruits	Cinnamic amides **(114–119)**	Inhibiting PL^Pro^	Song et al. (2014)
SARS-CoV	Isatis indigotica Fortune ex Lindl	Indigo **(120)**	Inhibits cleavage activity of the 3CL^pro^	Lin et al. (2005)
SARS-CoV	Lycoris radiata (L’Hér.) Herb. / ethanol	Lycorine **(121)**	Inhibits SARS-CoV in CPE inhibition assays	Li et al. (2005a), Lin et al. (2005b), Lin et al. (2005c)
SARS-CoV	Isatis indigotica Fortune ex Lindl	Aloeemodin **(122)**, Hesperetin **(55)**, Sinigrin **(123)**, Beta-sitosterol **(124)**	Inhibits cleavage activity of the 3CL^pro^	Lin et al. (2005)
SARS-CoV	Glycyrrhiza glabra L	Glycyrrhizin **(47)**	Inhibitor of replication	Cinatl et al. (2003)
SARS-CoV	*(In silico)*	Dieckol **(125)**	Inhibiting 3CL^pro^	Park et al. (2013)
SARS-CoV	*Psoralea corylifolia* L/ethanol	Psoralidin **(126)**	Inhibiting PL^pro^	Kim et al. (2014)
SARS-CoV	Rheum officinale Baill., Polygonum multiflorum Thunb. (Reynoutria multiflora (Thunb.) Moldenke)	Emodin **(127)**	Blocking the S protein and ACE2 interaction	Ho et al. (2007)
SARS-CoV	Cimicifuga rhizoma, Phellodendron cortex, Sophora subprostrata/methanol	—	Inhibitor of RNA synthesis and N and S expression *in vitro*	Kim et al. (2008)
MERS-CoV	*(In silico)*	Dihydrotanshinone **(128)**	Block virus entry, inhibit S proteins	Kim et al. (2018)
MERS-CoV	*(In silico)*	Dodecana **(129)**	Binding affinity to MERS-CoV	Rao et al. (2018)
MERS-CoV	*(In silico)*	Emetine **(130)**	Inhibiting the replication of MERS-CoV	Shen et al. (2019)

## Conclusion and Future Perspectives

At the present time no specific anti-COVID-19 drugs are available and research communities, countries, and public health organizations are investigating all means to combat this globally transmitted pandemic. Early implementation of strict physical distancing and sanitation protocols has played a role in reducing the incidence of COVID-19 in many parts of the world (Islam et al., 2020; Petersen et al., 2020). Evaluation of current promising antiviral agents is a necessary strategy to discover efficient treatments against this disease.

Natural compounds are widely recognized as complex structures, crafted by evolutionary processes to interact with macromolecular targets. Over the past years, investigations all over the world have generated renewed interest in the search for novel antiviral agents from plant origin. With consideration of the provided information on influenza viruses, SARS-CoV, and Mers-CoV, the new SARS-CoV-2 virus, which shows a broad clinical spectrum and dramatic expansion, might be controlled based on genomic organization similarities.

In this survey we summarized recently reported discoveries of natural compounds with activity against respiratory viruses. We introduced 130 natural compounds as possible therapies to fight against respiratory viruses, including influenza virus, SARS-CoV, MERS-CoV and SARS-CoV-2. Different groups of natural products have antiviral activities but the majority of these compounds belong to the alkaloid, flavonoid and terpenoid families. Although most of the natural compounds listed in this assay are potential inhibitors of COVID-19, the results are mostly based on theoretical or *in vitro* research. Most current research does not present analytical validation. Some natural products, including xanthoangelol E (**88**), hispidulin (**3**), quercetin (**6**), rutin (**8**), saikosaponin D (**36**), glycyrrhizin (**47**), methyl rosmarinate (**27**) and hesperetin (**55**) showed antiviral activities against different respiratory viruses *in vitro* and *in vivo.* These compounds can be recommended as potential lead candidates to prioritise for investigation. Additionally, resveratrol (**69**) is a natural compound which showed promising activity against COVID-19 in the clinical setting. In some cases the maximal suppression of virus infection and replication can be obtained by using a combination of drugs with different mechanisms of action. Natural products and herbal medicine can be considered as important drugs which can be used in combination with basic antiviral treatment.

Despite several existing reports about natural products and their antiviral activities, working with phytochemicals requires expertize and there are many challenges in working with natural compounds. For instance, the isolation, purification, and identification of the structures of these compounds might be paved with challenges. Also, the bioavailability of natural products should be considered before embarking on expensive clinical trials. Other issues include difficulties in prediction of a suitable dosage for these compounds, modes of drug delivery, and the outcome of the combination of two or more of these natural products. The flavonoid quercetin (**6**) is a highly promising compound on the basis of its antiviral activity against influenza A, SARS-CoV, and COVID-19. Future studies should focus on appropriate methods of delivery to combat respiratory viruses, such as nasal or throat sprays, and *in vivo* efficacy.

The structure-based drug design approach is recommended to allow synthetic chemists to develop effective anti-COVID-19 agents. In this regard, the relationship between structure and activity of the compound can be a viable strategy and guidance to create a broad range of anti-respiratory viral compounds. Some previously mentioned studies have given different perspectives, such as activity-guided fractionation, which can be used as a tactic to discover anti-COVID-19 medicines. Accordingly, screening programs may be a rational way to test traditionally used plants all over the world by working on a pseudovirus of COVID-19. Medicinal plants are known as a key natural resource for therapeutic agents. Whilst the future evolution of the current coronavirus pandemic remains unpredictable, beside the public health strategies there is an urgent need for global interdisciplinary cooperation between chemists, microbiologists, botanists and biochemists in order to find natural medicines against COVID-19 and to combat the current challenges, even during inter-epidemic periods.
